# Ainhum, a rare mutilating dermatological disease in a female Cameroonian: a case report

**DOI:** 10.1186/s12895-019-0092-6

**Published:** 2019-08-12

**Authors:** Diego Nitcheu Tchouakam, Joel Noutakdie Tochie, Marc Leroy Guifo, Simeon Pierre Choukem

**Affiliations:** 1 Health and Human Development (2HD) Research Network, Douala, Cameroon; 2 Bogo District Hospital, Bogo, Cameroon; 3 Departement of Surgery and Specialties, Faculty of Medicine and Biomedical Sciences, University of Yaoundé 1, Yaoundé, Cameroon; 4 Department of Surgery, University Hospital Center, Yaounde, Cameroon; 5 Department of Internal Medicine, Douala General Hospital, Douala, Cameroon; 6 Faculty of Medicine and Pharmaceutical Sciences, University of Dschang, Dschang, Cameroon

**Keywords:** Ainhum, Fifth toe, Cameroon

## Abstract

**Background:**

Ainhum is an idiopathic dermatological disease characterized by a progressive constricting ring usually on the fifth toe, which may lead to spontaneous auto-amputation of the affected toe. Timely diagnosis and treatment are the key elements to avert amputations with resultant mutilating deformities, permanent handicaps and psychological sequelae. Though common in African descents, this pathology has not been described in the Cameroonian literature. Herein, we report the case of an adult Cameroonian woman presenting with ainhum.

**Case presentation:**

A 54-year old Cameroonian was admitted to our primary healthcare centre with a 6-month history of a painful constriction band developing at the base of her right fifth toe. Her past history was uneventful. Based on the absence of trauma and spontaneous onset of the condition, the diagnosis of ainhum was most suggestive. She was managed surgically by excision of the band, disarticulated at right fifth metatarsophalangeal joint and skin closure. Her post-operative course after 1 year was uneventful.

**Conclusion:**

Here we presented a case of ainhum, a rare dermatological disease with few reports. In view of the serious complications of ainhum such as mutilating deformities with permanent physical disabilities and psychological trauma, we draw clinicians’ attention, especially wound care specialists to this rare but potentially handicapping disease, for timely diagnosis and management.

## Background

The word “Ainhum” derived from the Nago word (Brazil) meaning ‘fissure’ or the Yoruba word (Nigeria) meaning ‘to saw or cut’ is a relatively rare idiopathic skin disease [1]. This scarce dermatological pathology is characterized by an insidious development of a firm constricting band or ring on any phalange (finger or toe) leading to spontaneous auto-amputation, permanent deformities, physical handicaps and psychological trauma in severe cases [2–4]. Likewise, it may present as chronic fissuring, ulceration or infection at the base of the fifth toe [5]. It frequently affects the fifth toe and predominantly occurs in sub-Saharan Africans [3]. Timely diagnosis and treatment are crucial in preventing disease progression to mutilating deformities caused by auto-amputation of the affected phalenge [3].

A search of PUBMED with keywords: “ainhum”, and “Cameroon”, revealed no article. Herein, we present a female Cameroonian with ainhum. We describe the clinical features of this rare disease to facilitate timely recognition and appropriate management.

## Case presentation

A 54-year-old female from the Northern region of Cameroon, presented on January 12, 2018 to our primary health care hospital with a 6-month history of a painful circular constriction band at the base of her right fifth toe. There was no history of trauma, ligation or bangles on the toe nor laceration of the toe. Her past history was not notable for any chronic disease such as diabetes, ischaemic heart disease, peripheral vascular disease, skin pathologies, HIV/AIDS and psychiatric disorder. Also, she was a non-smoker. There was no family history of similar skin disease.

**Fig. 1:**
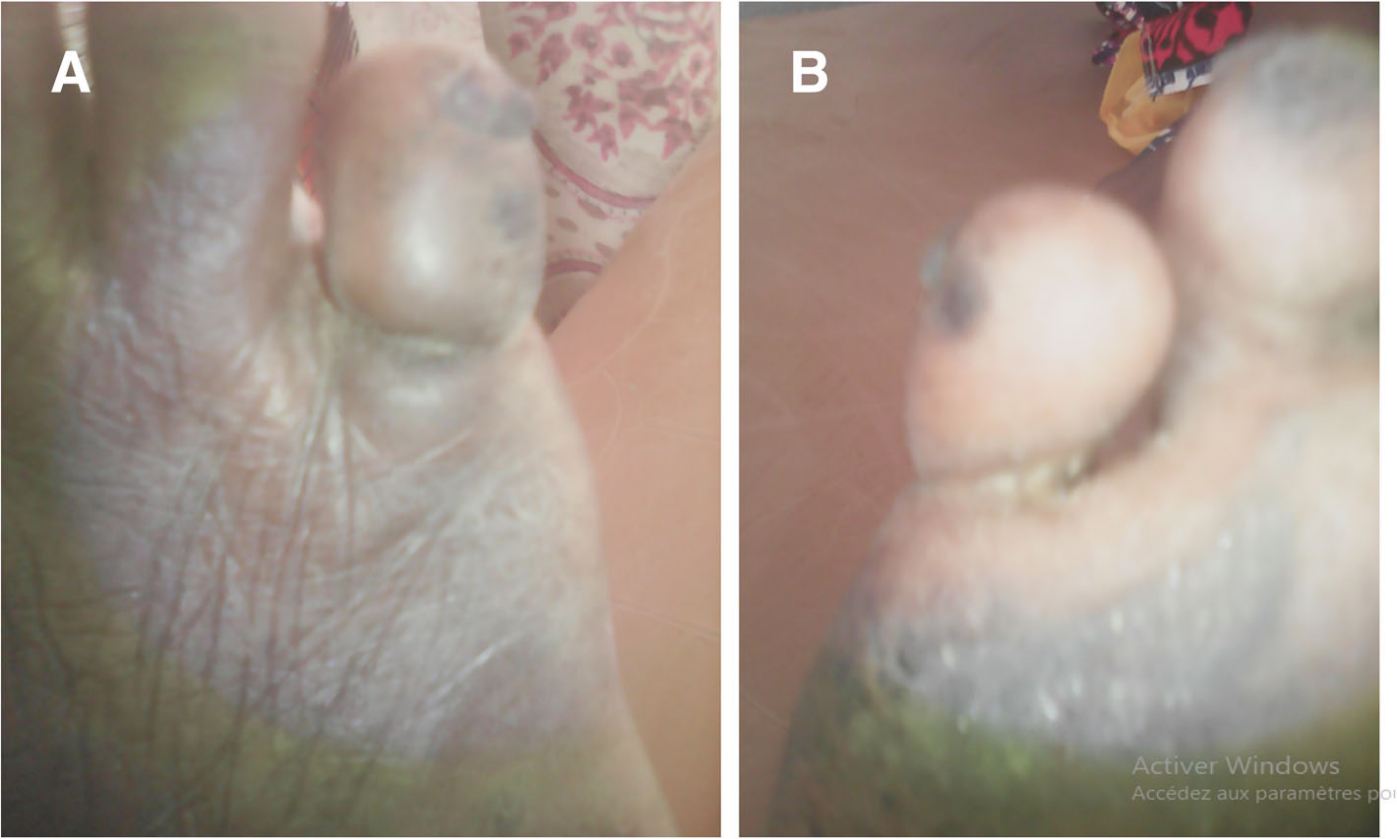
Dorsal view of the right fifth toe showing an annular constricting band at its base. The toe appears globular and oedematous (**a**), Plantar view of the right fifth toe showing the same annular constricting band posteriorly with plantar keratosis (**b**)

**Fig. 2:**
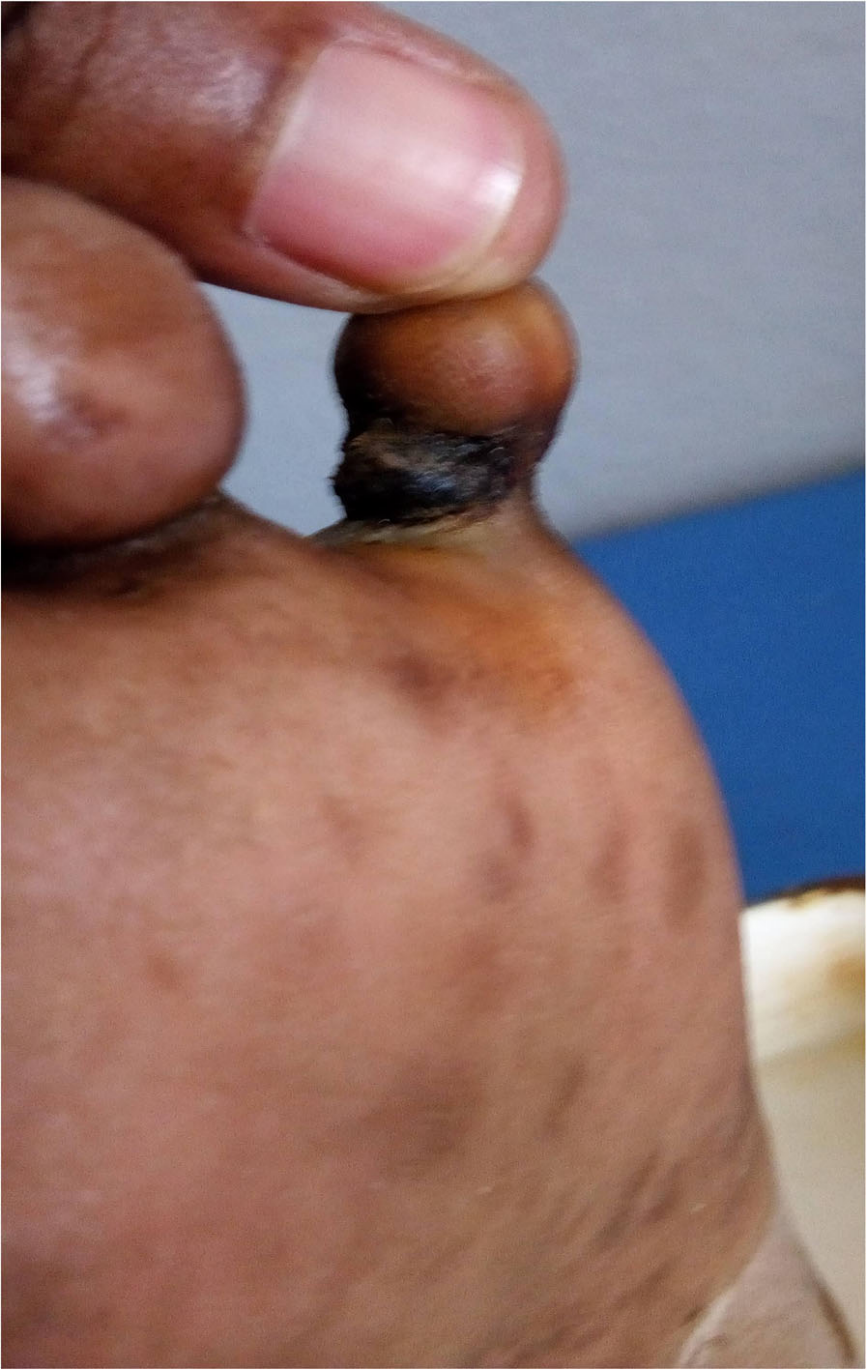
Plantar view of the right fifth toe showing an annular constrictingband at its base. The toe appears globular, oedematous and nearly amputated

On examination, she was conscious, oriented, and was not ill-looking. Her blood pressure was 138/74 mmHg, pulse rate 102 beats/minute and regular, respiratory rate 18 breaths/minutes and temperature 36.4 °C. All distal pulses were present and of normal volume. There was no regional lymphadenopathy. On examination of the right foot, the fifth had a circular constrictive band at the base of the proximal inter-phalangeal joint (see Fig. 1). A provisional diagnosis of a toe infection was made. She was admitted and administered cefuroxime 1.5 g/12 h intravenously (IV), metronidazole 500 mg/8 h IV, paracetamol 1 g/6 h IV, diclofenac 75 mg/ 12 intramuscularly and wound dressing every 48 h. Her clinical re-assessment 1 week later was notable for progression of the constricting band which made contact with the bone until a stage of near auto-amputation of its distal segment (see Fig. 2). The distal aspect of the toe was bulbous and oedematous. The affected toe was very mobile and any attempt to actively or passively mobilize it caused excruciating pain. There was bilateral plantar hyperkeratosis. The rest of her clinical assessment was normal. X-ray of the right foot could not be done because it was not available in our health facility. A laboratory panel requested on her admission revealed complete blood count (CBC): white blood cell count 8,400/mm^3^, haemoglobin 12.1 g/dl, platelet count of 348,000/mm3; raised C-reactive proteins at 48 mg/l and an erythrocyte sedimentation rate at 46 mm 1st hour and 89 mm 2nd hour. The worsening of her toe’s pathology prompted a quick diagnostic review, thereby advocating for the diagnosis of stage four ainhum with impending auto-amputation based on the history and clinical findings. After proper preoperative workups consisting of a complete blood count, prothrombin time and activated thromboplastin time (which were all normal), the constricting band was excised under local anaesthesia by infiltration of lidocaine 2% at its base, the right fifth toe was disarticulated at right fifth metatarsophalangeal joint and the skin was closed surgically. Histopathological examination of the amputated toe was not accessible. Her postoperative course was uneventful and she was discharged on day seven after wound healing. Follow-up to 1 year was equally uneventful.

## Discussion and conclusions

To the best of our knowledge, this is perhaps the first report of ainhum in Cameroon. The term “ainhum” was first described by a Brazilian physician called da Silva Lima in the year 1867 [6]. With regards to epidemiology, ainhum is an extremely rare dermatological disease with prevalence rates reported at 2.2% [1], 0.2% [7] and 0.015% [8] in Nigeria, Congo, and Panama, respectively. It has rarely been reported in Europe and Asia [3]. As such, ainhum may be easily overlooked because of its low frequency and variable clinical polymorphism, described below [9]. It has a preponderance for people of sub-Saharan African origin aged between 20 and 50 years [3]. Men are more predisposed than women with a male to female sex ratio of 2:1 [3]. Ainhum classically presents with bilateral involvement of the fifth toes in 75% of cases [10]. Noteworthy, a few cases of isolated finger [11] and isolated great toe involvements [12] have been reported. Generally, patients have a family history of ainhum [9]. These epidemiological facts concur with the above case who was a female of African descent and almost of the same age as the most affected age group. However, the patient differs from the previous epidemiological concepts by being a female, having a unilateral involvement and no family history.

Ainhum is commonly reported as an idiopathic disease [3]. Nonetheless, some authors suggested a few etiological hypotheses like infections (mycosis, mycobacteria), trauma, decreased vascular supply, peripheral neuropathy, and genetic (keratodermia) pathogeneses [2]. Cases related to trauma are often trivial, for instance walking barefoot in the tropics [3]. In the present case report, the absence of the aforementioned etiological causes supports the hypothesis of an idiopathic cause as previously described in the literature [3, 5]. Moreover, the diagnosis of ainhum is mainly clinical, described as a spot diagnosis by some authors [13]. The pathogenesis involves the development of a hard annular band from a flexural groove of the phalange, with gradual full circumferential constriction of the phalange [3]. The natural history progresses to spontaneous auto-amputation [3].

Although considered a spot diagnosis [13], established diagnostic criteria for ainhum entails the following three conditions: soft tissue constriction with bulbous enlargement of the toes, thinning of phalangeal bone and phalangeal lysis [1] which were all seen in the present case. It is worth to mention, that there are four clinical stages of ainhum [1]: 1) A clavus develops, which progresses to an annular fissure around the toe; 2) The toe becomes globular distal to the groove, associated with bone resorption and arterial narrowing; 3) The very painful bone separates at the joint with hypermobility of the toe; 4) A bloodless auto-amputation of the toe with severe pains. The indexed patient presented at stage 4, and we corrected her impending toe amputation surgically to ensure proper wound healing. Similarly to reports of Browne SG [7] and Morand L [2], ainhum was associated with plantar hyperkeratosis, implying that this association may be closely linked to the etiology of ainhum. Hence, like other authors [14, 15] we can consider that the indexed patient illustrated the hypothesis of idiopathic ainhum as a peculiar manifestation of a variety of hereditary plantar keratoderma affecting patients of African descent. Findings on x-ray imaging include a radiolucent ring constricting the base of the toe, bone resorption or osteolysis is seen in the distal and middle phalanges, with a characteristic tapering effect [16, 17]. An x-ray could not be performed owing to frequent resource-constrained settings described in low-income countries like ours [18]. The differential diagnosis of ainhum include pseudo-ainhum that occurs secondary to congenital annular bands which lead to constriction of digits described as Streeter’s dysplasia [19] or keratoderma hereditarium mutilans known as Vohwinkel syndrome [19], constrictions resulting from trauma or linked to other diseases like scleroderma, syphilis, leprosy, atypical keratoderma, diabetes mellitus, yaws, or vascular gangrene [13, 20]. Once more, infrastructural limitations hindered us from ruling out this differential diagnoses.

At early stages of ainhum, non-operative treatment using topical or injectable salicylate preparations [21], corticosteroids [22], or retinoids [3] is recommended. Surgical management for stage 1 and stage 2 ainhum entails a Z-plasty to release the constricting base of the toe has also been reported to yield favourable outcomes and avert toe amputation [1, 23, 24]. Surgical amputation is the mainstay of treatment of stages 3 and 4 [3], as was performed in the above case. Timely amputation helps in pain relief and prevention of surgical site infections [9]. If left untreated, auto-amputation, secondary infections, and locomotor imbalance may complicate ainhum [3].

The absence of radiographic imaging and histology of the amputated toe are important limitations of the present report. X-ray studies have been used in previous reports to confirm the diagnosis and the stage of ainhum [9, 17]. Also, histology examination was not performed in our case, but previous reports have shown thickening of the stratum corneum near the fibrous band, hyperkeratosis or acanthosis of the epidermis and the presence of lymphocytes and fibroblasts in the dermis in response to tissue damage and the chronic inflammation [21]. However, the diagnosis of the present case was limited to history and clinical examination given that ainhum is, first of all, a spot diagnosis [13] and imaging studies as well as histopathological examination help in ruling out differential diagnoses. Furthermore, this case report may be useful to draw attention to the occurrence of anihum in Cameroon, where it may be underdiagnosed.

In conclusion, we described a case of ainhum in a female patient in Cameroon, a dermatological mutilating condition, more common in sub-Saharan African males. The authors highlight the need for a high index of suspicion by healthcare providers especially wound care specialists, for timely diagnosis and management geared at preventing auto-amputations, permanent deformities and handicaps, psychological trauma and surgical site infections.

## Data Availability

Data sharing is not applicable to this article as no datasets were generated or analyzed during the current study.

## Abbreviations

IV: Intravenously
